# The Application of Ultrasonic Waves and Microwaves to Improve Antihyperglycaemic and Antimicrobial Activities of *Marrubium vulgare* Extracts

**DOI:** 10.3390/antibiotics11111475

**Published:** 2022-10-25

**Authors:** Aleksandra Gavarić, Jelena Vladić, Jelena Vujetić, Dragan Radnović, Ana Volarić, Jelena Živković, Katarina Šavikin, Senka Vidović

**Affiliations:** 1 Faculty of Technology, University of Novi Sad, 21000 Novi Sad, Serbia; 2 Institute of Food Technology, University of Novi Sad, 21000 Novi Sad, Serbia; 3 Faculty of Sciences, University of Novi Sad, 21000 Novi Sad, Serbia; 4 Institute for Medicinal Plants Research Dr. Josif Pančić, 11000 Belgrade, Serbia

**Keywords:** *Marrubium vulgare*, ultrasound extraction, microwave extraction, antihyperglycaemic activity, antimicrobial activity

## Abstract

Having scarce information about ultrasound assisted extraction (UAE) and microwave assisted extraction (MAE) of white horehound (*Marrubium vulgare* L.), the idea has emerged to determine the optimal process parameters for the maximization of polyphenols and to compare the efficiency of these green extraction technologies. The optimal UAE parameters are temperature of 73.6 °C, extraction time of 40 min and ultrasound power of 30.3 W/L, while the optimal MAE parameters are 63.8% ethanol, extraction time of 15 min and microwave power of 422 W. Extract obtained at optimal UAE parameters shows the highest antihyperglycemic activity (α-amylase inhibition: 50.63% and α-glucosidase inhibition: 48.67%), which can potentially be explained by the presence of chlorogenic acid and quercetin, which were not identified in the macerates. The most sensitive bacterial strain to optimal ultrasonic extract is *Bacillus cereus*, whereas the most sensitive fungal strain is *Saccharomyces cerevisiae*.

## 1. Introduction

White horehound (*Marrubium vulgare* L.) is a grey-leaved perennial herb belonging to the Lamiaceae family, distributed throughout the Eurasia and northern Africa zones [1]. There are contradictory literature data on the hypoglycemic activity of horehound. According to one source, aqueous horehound extract did not significantly reduce blood glucose levels [2], while according to another source, its administration induced a significant reduction in glucose levels in rats [3]. The variation in the results is most likely a consequence of the collection of plant material from different localities, which greatly affects the quality and quantity of the secondary metabolites, together with the use of different parts of the plant and different extraction methods. Several studies have shown that polyphenol-rich extracts can be a major cause of antihyperglycemic activity in animals and humans, most likely through the inhibition of α-amylase and/or α-glucosidase [4,5]. Oral hypoglycemic drugs used to treat diabetes have serious side effects, such as weight gain and gastrointestinal disorders [6]. Therefore, studies of potential insulin sensitizers, such as chlorogenic acid, which stimulate insulin action similar to the therapeutic action of metformin, are necessary [7]. 

The wide spectrum of solid/liquid extractions is available for the extraction and isolation of new functional ingredients. However, some common techniques are inconvenient due to inefficient time and elevated temperature, leading to the thermal degradation of phenolic compounds [8]. Therefore, employment of green extraction technologies is suitable to overcome these obstacles and improve the extraction yield and the quality of the obtained extracts. In line with that statement, some researchers investigated the application of a pulsed electric field as a pretreatment step for the intensification of the horehound essential oil isolation. They concluded that a pulsed electric field improves the extraction rate of the essential oil by 2–3 fold [9]. Another environment-friendly, efficient extraction technique is ultrasound-assisted extraction (UAE), the application of which has increased in recent decades due to the several disadvantages associated with conventional extraction techniques, such as high capital investment, large consumption of toxic organic solvents and their residues in the extract and energy expenditure. In addition, ultrasound is relatively easy to use, versatile, flexible and requires low investment as a result of a simple principle of UAE, which is based on cavitation effects causing the rupture of plant cell walls, thus increasing the contact area between the solid and solvent [10]. As opposed to traditional methods, in microwave assisted extraction (MAE), the heat and mass gradients act in the same direction (from inside outwards), with faster heating occurring inside the solids, where the dissolution takes place [11]. Microwaves generated by a magnetron interact with water and other polar molecules, causing their heating as the molecular dipoles try to orient themselves in the direction of the electromagnetic field. Part of electromagnetic radiation is thus converted into molecular motion and dissipated as heat. MAE affords much shorter extraction time, higher extraction yield and 5–10 fold less solvent consumption in comparison to conventional extraction [12].

In this study, the main goal was to identify the optimal UAE and MAE parameters responsible for enhanced extraction of polyphenols from horehound herba in order to deliver high-quality extracts with significant antihyperglycaemic and antimicrobial activities. The Box–Behnken experimental design, with three factors at three levels, was applied in order to provide exhaustive extraction of polyphenols and reduce the number of experimental runs.

## 2. Results and Discussion

### 2.1. Extraction Yield 

Among the recent research papers interested in the valorization of horehound extracts obtained by UAE and MAE, those focusing on marrubiin are predominant [13,14,15]. In this study, the extraction yield obtained by MAE varied in the range 13.74–22.61%, and was slightly higher than the yield in the UAE, which varied from 12.91 to 20.08%. Compared with the extraction yield in the maceration with 50% ethanol, i.e., MAC (11.55%), both UAEopt (17.92%) and MAEopt (16.30%) extraction yields, were higher. Other researchers also applied Soxhlet extraction (18 h) of the aerial parts of horehound with ethanol as solvent and obtained a yield of 11.27% [13]. As opposed to 18 h of extraction, 6.22 min of MAE provided a yield of 20.48%. In addition, the S/L ratio in MAE was optimized at 1:32 (m/V), suggesting lower solvent consumption compared to Soxhlet extraction (1:50, m/V). Another study concluded that the most favorable MAE conditions in terms of extraction yield were temperature of 40 °C, 20% ethanol as a solvent and extraction time of 15 min [14]. The extraction yield (14.2%) and concentration of marrubiin (0.91%) were significantly improved at the optimized UAE conditions (ultrasound power 467 W, extraction time of 47 min and S/L ratio 1:33, m/V) as compared to the conventional method [15].

### 2.2. Total Phenolic Content

Experimentally obtained TP in horehound extracts generated by UAE varied from 73.66 to 99.30 mg GAE/g, depending on the applied extraction conditions. In our study, the lowest TP yield was observed after 60 min of extraction at a temperature of 40 °C and at an ultrasonic power of 60 W/L. Generally, low TP yields (73.66–83.89 mg GAE/g) were obtained using temperature of 40 °C, while higher TP yields (90–99.30 mg GAE/g) were obtained using 80 °C. The highest TP yield (99.30 mg GAE/g) in horehound extract was obtained at 80 °C after 40 min of extraction and at an ultrasound power of 42 W/L. These results are in accordance with a previous study, in which it was reported that the highest TP in sage ultrasonic extract was also obtained at 80 °C, while all UAE extracts obtained at 40 °C had significantly lower TP [16]. The highest TP yield from wild garlic was obtained at 80 °C using the same 50% ethanol concentration [17]. This suggests that temperature could be appointed as the most influential parameter in UAE. 

In the case of MAE, TP varied in the range 81.94–117.58 mg GAE/g. It could be confirmed that both UAE and MAE ensured a remarkable increase in TP yield compared to S/L extraction with 30, 50 and 70% ethanol (63.77 mg GAE/g, 73.68 mg GAE/g and 70.90 mg GAE/g, respectively). The highest TP yield was obtained using 50% ethanol for 25 min of extraction at a microwave power of 600 W. Comparing the maximal TP yields obtained by UAE and MAE, it could be seen that MAE provides 15% higher TP. This difference could be attributed to the intense cell destruction provided by MAE. In another study, 80% methanol was used for MAE of horehound leaves, setting the process parameters as follows: temperature of 60 °C, extraction time of 10 min and microwave power of 800 W. The TP of 6.02 mg GAE/g was determined to be significantly lower than the TP (86.05 mg GAE/g) obtained in the present study for 15 min of extraction at 800 W [1].

### 2.3. Total Flavonoid Content 

Experimentally obtained values of TF observed in horehound extracts created by UAE and MAE exploitation are presented in Table 1 and Table 2, respectively. The TF in horehound extracts obtained by UAE ranged between 44.59 and 53.19 mg CE/g, which is slightly higher than the TF yield (42.51 mg CE/g) obtained by S/L extraction lasting for 24 h. The highest TF was obtained at 80 °C after 40 min extraction at an ultrasound power of 42 W, as was the case with TP, suggesting a solid correlation between the influence of UAE parameters and the monitored responses, TP and TF.

The TF observed in MAE extracts ranged between 49.76 and 65.80 mg CE/g. Again, the TF yield in MAE was slightly higher than in UAE, replicating the pattern noticed in TP. The maximal TF in MAE was obtained using 70% ethanol for 35 min of extraction at microwave power of 600 W. A TF yield of 45.21 mg CE/g was obtained at a temperature of 60 °C, extraction time of 10 min and microwave power of 800 W [1]. This is slightly lower than the TF yield (54.70 mg CE/g) obtained in present work for 15 min of extraction at 800 W.

### 2.4. Antioxidant Activity

The antioxidant properties of horehound methanol extracts were investigated using DPPH assay and the results revealed a strong activity with IC_50_ value in the range 8.24–12.42 μg/mL [18]. The IC_50_ value for horehound was reported to be 0.0386 mg/mL [1], which is twice lower than the IC_50_ value (0.017 mg/mL) obtained in present study for 15 min of extraction at 800 W. Others used a central composite design to optimize the MAE of horehound in order to maximize its antioxidant activity. The optimized conditions were microwave power of 539 W, irradiation time of 373 s and S/L ratio of 1:32 (m/V). They reported an IC_50_ value of 0.066 mg/mL [13], which is out of the IC_50_ value interval (0.027–0.013 mg/mL) obtained in seventeen MAE runs conducted in our study. 

### 2.5. Model Fitting

Experimental results of the investigated responses (Y, TP, TF, IC_50_ and EC_50_) obtained under different UAE (temperature, extraction time, ultrasonic power) and MAE (extraction time, ethanol concentration, ultrasonic power) conditions using BBD are presented in Table 1 and Table 2, respectively. 

Results were fitted to a second-order polynomial model (Equation (13)) and multiple regression coefficients were generated for all five responses using the method of least squares. Analysis of variance (ANOVA) was employed in order to check the fitness of the applied models and the *p*-values of regression coefficients for each investigated response are summarized in Table 3.

The coefficient of multiple regression (*R*^2^) was used as the first indicator of the model adequacy. Descriptive statistics was in accordance with ANOVA, since *R*^2^ was particularly high for Y, TP, TF, IC_50_ and EC_50_ (0.9668, 0.8349, 0.8717, 0.9567, 0.8304, respectively) obtained by UAE. The similar situation was observed with Y, TP, TF, IC_50_ and EC_50_ obtained by MAE, where the observed *R*^2^ were 0.9141, 0.9334, 0.8386, 0.8337 and 0.8302, respectively. The relatively low (<12%) coefficient of variance for all responses suggested good reproducibility of the investigated systems. Good adequacy of the polynomial models for UAE indicated by descriptive statistics was confirmed by highly significant *p*-values (<0.001) for extraction yield and IC_50_, while it was approved by significant *p*-values (<0.05) for TP, TF and EC_50_ (Table 3). Good adequacy of the polynomial models for MAE indicated by descriptive statistics was also confirmed by highly significant *p*-values (<0.01) for extraction yield and TP, while it was approved by significant *p*-values (<0.05) for TF, IC_50_ and EC_50_ (Table 3). Moreover, proper model fitness has also been confirmed by insignificant lack of fit (*p* > 0.05) for all of the applied models. Method of least squares was used for calculation of regression coefficients in Equation (13), which provided descriptive model equations (Equations (1)–(10)), obtained by UAE and MAE:

Y = 16.15 + 2.32X_1_ + 0.78X_2_ + 0.62X_3_ (1)

TP = 0.083 + 7.97·10^−3^ X_1_ + 4.14·10^−3^ X_1_^2^ (2)

TF = 0.051 − 1.40·10^−3^ X_2_ + 2.40·10^−3^ X_1_X_3_ − 1.86·10^−3^ X_1_^2^ (3)

IC_50_ = 0.017 + 5.84·10^−4^ X_3_ + 1.58·10^−3^ X_1_X_3_ − 1.40·10^−3^ X_1_X_3_ + 3.33·10^−3^ X_1_^2^ + 8.51·10^−4^ X_3_^2^ (4)

EC50 = 0.058 + 4.64·10^−3^ X_1_ + 2.49·10^−3^ X_3_ − 3.07·10^−3^ X_1_^2^ (5)

Y = 18.62 − 3.1X_2_ + 1.04X_3_ (6)

TP = 0.083 + 8.36·10^−3^ X_2_ − 8.87·10^−3^ X_3_ + 5.91·10^−3^ X_1_^2^ + 5.87·10^−3^ X_2_^2^ + 6.72·10^−3^ X_3_^2^ (7)

TF = 0.054 + 4.71·10^−3^ X_2_ − 2.82·10^−3^ X_3_ + 3.36·10^−3^ X_2_^2^ (8)

IC_50_ = 0.019 + 1.77·10^−3^ X_1_ + 2.83·10^−3^ X_2_ + 2.89·10^−3^ X_1_X_2_ (9)

EC50 = 0.067− 0.011X_2_ (10)

where, X_1_, X_2_ and X_3_ are temperature, extraction time and ultrasonic power in the case of UAE (Equations (1)–(5)), while they represent extraction time, ethanol concentration and microwave power in the case of MAE (Equations (6)–(10)). Predictive model equations (Equations (1)–(10)) interpreted reduced Equation (13), since the coefficients of variables with inferior influence could be neglected.

### 2.6. Process Parameters Influencing UAE and MAE 

**Figure 1:**
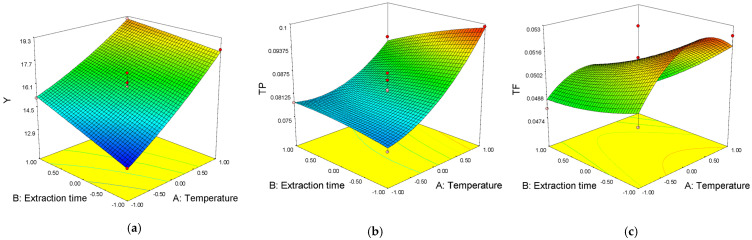

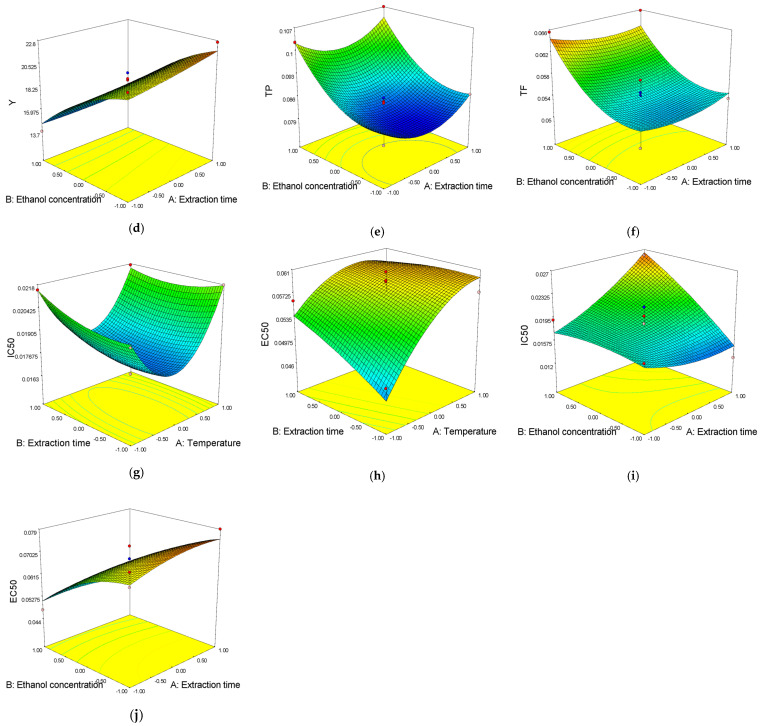
Response surface plots showing combined effects of temperature and extraction time on Y, TP, TF, IC_50_ and EC_50_ in UAE extracts (**a**–**c**,**g**,**h**) and combined effects of extraction time and ethanol concentration on Y, TP, TF, IC_50_ and EC_50_ in MAE extracts (**d**–**f**,**i**,**j**).

In UAE, the linear temperature term (*p* < 0.0001) is dominant over other parameters in the context of the influence on the extraction yield. According to the degree of influence, the second most dominant is a linear term of the extraction time, which showed a very significant (*p* < 0.01) influence on the extraction yield (Figure 1). In MAE, only the linear term of the ethanol concentration (*p* < 0.001) showed a markedly significant effect on the extraction yield (Figure 1). According to the ANOVA results from Table 3, it can be observed that in the UAE, the linear temperature term (*p* < 0.01) shows a significant effect, while the square temperature term (*p* < 0.1) shows a moderately significant effect on TP.

In the case of MAE, the most influential parameters were the linear terms of ethanol concentration and microwave power (*p* < 0.001), while their quadratic terms had a significant effect (0.01 < *p* < 0.05) on TP. In the case of UAE, the linear term of the extraction time (*p* < 0.1) showed a moderately significant effect on TF, while the linear member of the interaction between temperature and ultrasound power showed a markedly significant effect (*p* ˂ 0.005). In MAE, both the linear and square terms of ethanol concentration (*p* < 0.1) showed a moderately significant effect on the TF.

### 2.7. Phenolic Acids

In the extracts obtained by maceration, the prevalent flavonoid compounds are the glycosides quercetin, rutin and hyperoside. The content of rutin alters in range 20.41–236.93 μg/mL, while hyperoside varies in range 2.71–25.58 μg/mL. The most dominant compound in all macerates is rutin. The relatively low content of ferulic acid (8.39 µg/mL), rutin (30.63 µg/mL) and hyperoside (5.78 µg/mL) in MAC-W extract can be explained by the use of water as a solvent, which has low selectivity. The MAC showed the highest content of ferulic and *p*-coumaric acid, as well as rutin and hyperoside, compared to other macerates (Table 4). 

Chlorogenic acid and quercetin were not detected in any of the tested macerates. The eight phenolic acids and five flavonoids were identified in the ethyl acetate fraction of horehound [19]. Among the flavonoid aglycones present in the ethyl acetate fraction, apigenin, luteolin, kaempferol, quercetin and hyperoside were the most dominant. The detected phenolic acids were caffeic, ferulic and *p*-coumaric acid. The contents of caffeic (0.12 mg/g), ferulic (0.52 mg/g) and *p*-coumaric (0.06 mg/g) acids are significantly lower than their contents in MAC (caffeic acid (1.10 mg/g), ferulic acid (2.17 mg/g) and *p*-coumaric acid (1.85 mg/g)). According to the HPLC results (Table 4), UAEopt proved to be richer in phenolic acids compared to MAEopt, with a higher content of chlorogenic acid as well as the flavonoid rutin. 

If the chemical profiles of UAEopt, MAEopt and MAC are compared, it can be concluded that UAEopt and MAEopt contain significantly fewer cinnamic acid derivatives and are richer in chlorogenic acid (UAEopt: 33.11 μg/mL and MAEopt: 23.23 μg/mL) and quercetin (UAEopt: 34.88 μg/mL and MAEopt: 30.05 μg/mL), which were not identified in the macerates. The content of rutin in UAEopt (49.59 μg/mL) and MAEopt (34.01 μg/mL) is significantly lower compared to that in MAC (236.93 μg/mL), most likely due to the hydrolysis of rutin to its aglycone quercetin, using these extraction techniques. In that context, some researchers examined the stability of 40 phenolic compounds in the ultrasonic extract obtained at temperature of 70 °C and concluded that rutin degradation (22.3%) and *p*-coumaric acid degradation (10.2%) occurred [20]. The least degradation is present in hyperoside whose contents in UAEopt (19.06 μg/mL) and MAEopt (14.71 μg/mL) extracts are similar to each other and slightly lower than MAC.

Others examined the degradation of seven phenolic acids under the influence of ultrasound in various commonly used extractants. Ferulic and *p*-coumaric acids have been shown to be stable in all extractants, while caffeic has undergone degradation, and the degree of degradation varies depending on the extractant used. The concentration of caffeic acid in ethanol extract after the UAE was reduced by 8.9%, while in the aqueous extract, the concentration was reduced by only 1%. From these results, it become apparent that the stability of phenolic acids in the UAE is also affected by the type of extractant used [21]. In this study, the chlorogenic acid content in UAEopt (1.84 μg/mg) and MAEopt (1.43 μg/mg) extracts is lower than its content in the aqueous extract [3]. Although the UAE is often used to extract phenolic acids due to higher extraction yields and shorter extraction time, the cavitation effect may be responsible for the degradation of flavonoids and carotenoids. A slight degradation of rutin has been observed in the UAE, although this compound possesses a sugar moiety that can potentially protect the molecule from degradation. Rutin degradation using UAE has also been previously described, and this phenomenon has been explained by the reaction of rutin with highly reactive hydroxyl radicals formed during UAE in a water-containing solvent, while glycosides were stable under the given conditions [22].

In MAE, the influences of a different microwave power (160–500 W) and an extraction time (1–10 min) on flavonoid degradation were examined. The increase in microwave power and time caused greater degradation of these compounds. The least degradation was observed for rutin and naringin, while significant degradation was observed for myricetin, kaempferol, ramnetin and quercetin. In this study, the extraction time of 15 min and the microwave power of 422 W caused an intensification of the extraction process due to higher pressure and temperature. Elevated temperature simultaneously results in improved extraction efficiency and a degradation of thermolabile compounds, which is indicated by a weaker profile of phenolic compounds compared to UAEopt. The stability of 22 phenolic compounds was investigated during the MAE with methanol as a solvent in the temperature range 50–175 °C and it was concluded that caffeic, *p*-coumaric and ferulic acids are stable at temperatures up to 100 °C, while at 125 °C they are subject to significant degradation. Of the cinnamic acid derivatives the most stable compound of the tested is ferulic acid, which is subject to degradation of 10.9% at 150 °C, while caffeic acid suffered a degradation of 16.3% at the same temperature. The most stable compound with the least number of substituents was found to be *p*-coumaric acid, which did not undergo degradation at 175 °C [23]. Also, in this work, *p*-coumaric acid underwent a lower degree of degradation compared to ferulic and caffeic acids.

### 2.8. Process Optimization

In this research, in the case of UAE, the optimal process parameters for investigated responses (Y, TP, TF, IC_50_ and EC_50_) were temperature of 73.6 °C, extraction time of 40 min and ultrasound power of 30.3 W/L. Whilst in the case of MAE, the optimal process parameters for all five responses were ethanol concentration of 63.8%, extraction time of 15 min and microwave power of 422 W. It could be observed from Table 5 that MAE ensures slightly higher yields of polyphenols, while antioxidant activities, expressed by two in vitro assays, of both UAEopt and MAEopt, are rather similar. 

In order to confirm the predictive mathematical models, validation was performed by separate extractions at optimal parameters for UAE and MAE. According to the results, it could be concluded that the experimentally obtained values were in line with the predicted results.

### 2.9. Antihyperglycaemic Activity 

**Figure 2:**
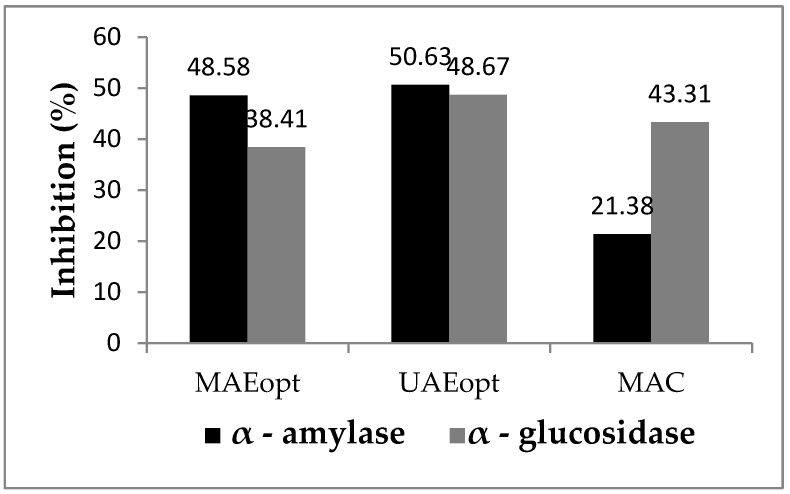
Inhibitory potential of horehound extracts for α-amylase and α-glucosidase.

In this study, in vitro inhibitory potential of horehound ethanolic extracts (UAEopt, MAEopt and MAC) for the enzymes α-amylase and α-glucosidase was investigated. The inhibitory potential of horehound extracts for α-amylase has the following order: UAEopt > MAEopt > MAC (Figure 2). According to the results, UAEopt and MAEopt extracts show similar inhibitory activity against α-amylase (50.63 ± 0.05% and 48.58 ± 0.04%), which is significantly higher compared to MAC (21.38 ± 0.05%). Total α-amylase inhibition expressed via acarbose equivalent for UAEopt, MAEopt, and MAC is 81.37; 75.69 and 0.33 μg/mL, respectively. It can be concluded that UAEopt inhibits α-amylase the same order of magnitude as acarbose (80 μg/mL).

The investigated UAEopt, MAEopt and MAC differ significantly in their chemical compositions, with UAEopt and MAEopt being richer in chlorogenic acid and quercetin. It was also shown that chlorogenic acid possesses antihyperglycemic potential in rats with induced diabetes [24]. Chlorogenic acid is known to lower blood glucose and inhibit glucose-6-phosphatase, a key enzyme that catalyzes the final step of glycogenolysis and gluconeogenesis, the two major metabolic pathways responsible for the release of glucose from the liver [25]. Previous experimental data show that chlorogenic acid stimulates glucose uptake into liver cells and regulates excessive glucose production by inhibiting this enzyme, thus controlling glycemic status in type 2 diabetes. Quercetin is also recognized as a flavonol that improves glycemic control and reduces diabetic nephropathy [26]. This aglycone of rutin reduces blood glucose concentrations and increases insulin release in rats with induced diabetes [27]. 

The inhibitory potential for α-glucosidase is weaker compared to α-amylase and has the following order: UAEopt > MAC > MAEopt (Figure 2). As in the case of α-amylase, UAEopt shows the highest antihyperglycemic activity (48.67 ± 0.04%), which can potentially be explained by the presence of chlorogenic acid (33.11 μg/mL) and quercetin (34.88 μg/mL). The total inhibition of α-glucosidase expressed via acarbose equivalent for UAEopt, MAEopt and MAC is 26.10; 18.60 and 22.18 μg/mL, respectively. It can be concluded that UAEopt inhibits α-glucosidase by the same order of magnitude as acarbose (27 μg/mL).

### 2.10. Antimicrobial Activity

The problem of microbial resistance continues to rise, despite the fact that there is a wide palette of antibiotics on the market, due to the uncontrolled use of these drugs against many infectious diseases [28]. Horehound essential oil is already recognized as a potential antimicrobial agent, but other extracts are still not identified. In order to gain an insight into the antimicrobial activity of horehound ethanolic extracts, minimum inhibitory concentration (MIC) values were determined for eleven pathogens. The differences in MIC values obtained for Gram-positive (Gr^+^) and Gram-negative (Gr^−^) bacterial strains are shown in Table 6.

It was shown that Gr^+^ bacteria are more sensitive to the action of all three extracts compared to Gr^−^ bacteria because MIC values for Gr^+^ vary in the range 3.13–12.50 mg/mL, and for Gr^−^ in the range 12.50–25.00 mg/mL. The different susceptibilities of Gr^+^ and Gr^−^ bacteria are a consequence of the different structure of the cell wall. Gr^−^ bacteria are surrounded by a thin layer of peptidoglycan encircled by an outer membrane made up of lipopolysaccharides, while Gr^+^ bacteria lack an outer membrane. Thus, the outer membrane represents a barrier that limits the diffusion of active ingredients [29]. The most sensitive bacterial strain to UAEopt and MAEopt extracts is *Bacillus cereus*, which is confirmed by the MIC value (3.13 mg/mL) which is twice lower than the one for MAC. 

In the case of antifungal activity, the essential oil of horehound was tested on four strains of fungi, while horehound methanol extract was tested on *Candida albicans* [30]. Horehound methanol extract showed moderate to significant antibacterial activity in five out of the six bacterial organisms tested compared to standard ciprofloxacin. The study showed that the horehound methanol extract is very effective against *B. subtilis*, *S. epidermidis*, *S. aureus* (Gr^+^) and *C. albicans*, and moderately effective against *P. vulgaris* and *E. coli*, while in the case of *P. aeruginosa* (Gr^−^), it is inefficient. The lowest MIC value of 100 mg/mL was achieved in most of the tested bacteria (*B. subtilis*, *S. aureus* and *S. epidermidis*) and in the fungus *Candida albicans*, while the highest MIC of 400 mg/mL was achieved in *E. coli* and *P. vulgaris,* which showed moderate sensitivity [31]. In this study, the MIC values (12.50 mg/mL) of UAEopt and MAEopt for strains of *B. subtilis*, *S. aureus* and *S. epidermidis* are about 8-fold lower than the MIC values of methanol extract of horehound, indicating that UAEopt and MAEopt are significantly more potent for these strains.

One of the selected strains for testing the antimicrobial activity of UAEopt and MAEopt is *S. aureus*. The results showed that this strain was equally sensitive to UAEopt, MAEopt and MAC (MIC = 12.5 mg/mL). In the case of yeasts, *Saccharomyces* is multiple times more sensitive (MIC ≤ 0.05 mg/mL) to the action of UAEopt and MAEopt compared to *Candida albicans*, while inhibition of *S. cerevisiae* growth requires about 4-fold higher MAC concentration (MIC = 0.20 mg/mL). The *Saccharomyces* is considered an important industrial microorganism widely used in the production of food and alcoholic beverages, as well as being a source of yeast-derived β-glucans. Despite its numerous beneficial applications, *S. cerevisiae* can also act as a human opportunistic pathogen causing a variety of infections, especially in immunocompromised individuals [32]. The minimum bactericidal concentration (MBC) values were also determined for eleven pathogens (Table 6). In the case of MAC, the MBC value is at least twice as high as the MIC for a given bacterial or fungal strain. In the case of UAEopt, MIC and MBC have the same values for the following Gr^−^ strains: *E. coli* (25 mg/mL), *Proteus mirabilis* (25 mg/mL) and for the yeast *Saccharomyces* (≤0.05 mg/mL). In the case of MAEopt, MIC and MBC have the same values for the following Gr− strains: *E. coli* (25 mg/mL), *Klebsiella pneumoniae* (25 mg/mL), *Pseudomonas aeruginosa* (25 mg/mL) and for the yeast *Saccharomyces* (≤0.05 mg/mL). 

## 3. Materials and Methods

### 3.1. Plant Material

*M. vulgare* was bought from the local supplier of cultivated plants “Chamomilla” (Banatski Karlovac, Serbia). The aerial parts of *M. vulgare* were air-dried in thin layers, collected in the paper bags, and stored at a room temperature. Afterwards, the dried *M. vulgare* herba was grounded in a domestic blender and the mean particle size (0.28 mm) of herbal material was determined using vibration sieve sets (CISA, Cedaceria, Spain).

### 3.2. Chemicals

Reagents used in the methods, 1,1-Diphenyl-2-picryl-hydrazyl-hydrate (DPPH), Folin-Ciocalteu and (±)-Catechin were purchased from Sigma (Sigma-Aldrich Chemie GmbH, Sternheim, Germany). The following reagents were also purchased from Sigma-Aldrich Chemie: iron (III)-chloride, potassium hexacyanoferrate (III), sodium hydrogen phosphate anhydrous, sodium dihydrogen phosphate and trichloroacetic acid. Gallic acid was purchased from Sigma (St. Luis, MO, USA). α-Amylase from porcine pancreas, α-glucosidase from *Saccharomyces cerevisiae*, 4-Nitrophenyl α-d-glucopyranoside and acarbose were obtained from Sigma (St Louis, MO, USA). All other chemicals and reagents were of analytical grade.

### 3.3. Conventional Solid/Liquid Extraction

Conventional solid/liquid (S/L) extraction was performed in order to determine experimental domain for the ethanol concentration used in the optimization scheme and to compare the yields of polyphenols obtained by conventional and green extraction techniques. The S/L ratio was 1:10. Distilled water and different concentrations of ethanol (30, 50 and 70%) were used. Extractions were performed in a shaker with temperature control (KS 4000i, IKA, Staufen, Germany) at 25 °C for 24 h with 150 rpm shaking speed. After extraction, the obtained extracts were filtrated through filter paper. The extracts were collected into glass vials and stored at 4 °C prior to analysis.

### 3.4. Ultrasound-Assisted Extraction (UAE)

In all UAE experimental runs, 10 g of sample was mixed with 100 mL of 50% ethanol in 250 mL glass flasks. Selection of optimal ethanol concentration (50%) was based on the highest polyphenol yield obtained by conventional extraction performed with a wider range of ethanol concentrations (30, 50, 70 and 96%). UAE was performed in a sonication water bath (EUP540A, Euin-struments, France). Temperature (40, 60 and 80 °C), extraction time (40, 60 and 80 min) and ultrasonic power (24, 42 and 60 W/L) were independent variables which were set by the control panel of the instrument. In order to prevent evaporation of the extraction solvent, condenser was added on the flask during extraction. Flasks were always positioned at the same distance from the transducer in the ultrasonic bath in order to provide constant ultrasonic power. After extraction, extracts were filtered through filter paper, collected into glass vials, sealed and stored at 4 °C prior to analysis.

### 3.5. Microwave-Assisted Extraction (MAE)

Mono-mode MAE was performed in a homemade setup consisting of a microwave oven (NN-E201W, Panasonic, Osaka, Japan) and appropriate glass apparatus with round flask attached to a condenser. In all MAE experimental runs, 10 g of horehound was mixed with 100 mL of extraction solvent in 250 mL round glass flasks. The flask was then placed in the microwave oven and extraction was performed. Flasks were always positioned at the same distance from the magnetron. Ethanol concentration (30, 50 and 70%), extraction time (15, 25 and 35 min) and irradiation power (400, 600 and 800 W) were independent variables. After the extraction, crude extracts were filtered through filter paper under vacuum, collected into glass vials, sealed and stored at 4 °C prior to analysis.

### 3.6. Analysis of Bioactive Compounds

#### 3.6.1. Total Phenols Content

The content of total phenolic compounds (TP) in horehound extracts was determined by the Folin–Ciocalteu procedure [33]. The absorbance was measured at 750 nm. TP was expressed as mg of gallic acid equivalent per g of dry weight of extract (mg GAE/g). All experiments were performed in triplicate.

#### 3.6.2. Total Flavonoids Content

The total flavonoids content (TF) was determined using aluminum chloride colorimetric assay [34], using catechin as a standard compound. The absorbance was measured at 510 nm. The content of total flavonoids was expressed as mg of catechin equivalent per g of dry weight of extract (mg CE/g). All experiments were performed in triplicate.

#### 3.6.3. DPPH Assay

The free radical scavenging activity of horehound extracts was determined using a simple and fast spectrophotometric method previously described elsewhere [35]. The absorbance was measured at 517 nm. Radical scavenging capacity (RSC (%)) was calculated according to Equation (11). Antioxidant activity was expressed as IC_50_ value, which represents the concentration of extract solution required for obtaining 50% of radical scavenging capacity.

%RSC = 100 − ((A_sample × 100))/A_control(11)

where, A_sample is the absorbance of sample solution and A_control is the absorbance of control. All experiments were performed in three replicates.

#### 3.6.4. FRAP Assay 

The reducing power of horehound extracts was determined by a method previously described in the literature [36]. Various concentrations of extracts obtained were mixed with phosphate buffer (2.5 mL, 0.2 M, pH 6.6) and 2.5 mL of 1% potassium ferricyanide (K_3_Fe(CN)_6_). The mixture was incubated for 20 min at 50 °C. After incubation, 2.5 mL of a 10% trichloroacetic acid solution was added to the mixture and the mixture was centrifuged (Tehtnica Železniki, Slovenia) for 10 min at 3000 rpm (0.402× *g*). The obtained supernatant (2.5 mL) was mixed with bidestillated water (2.5 mL) and 0.1% FeCl_3_ solution (0.5 mL). Absorbance was measured at 700 nm. Antioxidant activity was expressed as EC_50_ value (mg/mL), which represents the concentration of extract solution required for 50% reduction of Fe^3+^ ions in reaction mixture. All experiments were performed in triplicate, and results are expressed as mean values.

#### 3.6.5. HPLC Analyses of Phenolic Acids

The content of phenolic acids in horehound extracts obtained at optimal UAE parameters (UAEopt) and at optimal MAE parameters (MAEopt) was analysed by Agilent 1200 Series HPLC with DAD detector (Agilent Technologies, Palo Alto, CA, USA) equipped with LiChrospher^®^ 100 RP-18 column (250 × 4 mm, 5 μm). The binary mobile phase consisted of 0.17% water solution of formic acid (A) and acetonitrile (B). The injection volume of the sample was 10 μL and flow rate was 0.8 mL/min, while elution was performed with a gradient according to the following scheme: 0–53 min, 0–100% B. The detection wavelength was set at the range of 200–400 nm for phenolic acids. The examined samples were analyzed in three independent replicates. Identification of compounds was performed based on comparison of retention times and absorption spectra of unknown peaks with reference standards.

### 3.7. Antihyperglycaemic Activity

#### 3.7.1. α-Amylase Inhibitory Potential (α-AIP)

The α-amylase inhibition assay was performed according to the relevant procedure [37] with minor modifications. Acarbose (a generic antidiabetic drug) was used as a positive control that inhibited α-amylase in a dose-dependent manner, providing a value of 80 μg/mL. Briefly, 100 µL of sample, positive control (2 mM acarbose) or negative control (distilled water) was added to 200 µL of α-amylase solution in 0.02 M sodium phosphate buffer (pH 6.9). The tubes were incubated at room temperature for 5 min. Thereafter, 200 µL of 1% starch solution (prepared in the same buffer) was added to each tube and incubated for 6 min. Finally, 100 µL of dinitrosalicylic acid (dye reagent) was added, and the tubes were placed in a water bath at 100 °C and incubated for 5 min. The distilled water (1600 µL) was added to the mixture, and an absorbance was measured at 540 nm (T80 UV-Vis Spectrophotometer, PG Instruments LTD, Leicester, UK) in triplicate. The percentage of inhibition was calculated according to the following equation:

%Inhibition = (∆A_control − ∆A_sample)/∆A_control × 100(12)

where, ∆A_control and ∆A_sample are differences in absorbance of the control and sample.

#### 3.7.2. α-Glucosidase Inhibitory Potential (α-GIP)

The test for this method was previously described elsewhere [38]. Acarbose (positive control) inhibited α-glucosidase in a dose-dependent manner, providing a value of 27 μg/mL. In the cells of the microtiter plate (12 × 8), 100 µL 2 mmol/L 4-nitrophenyl-α-d-glucopyranoside in 10 mmol/L potassium phosphate buffer (pH 7.0) and 20 µL sample were measured. The reaction was initiated after the addition of 100 µL of enzyme solution. In parallel with the samples, control and blank samples were prepared and analyzed. Sample microtiter plates were incubated for 10 min at 37 °C, after which, the absorbance was measured at 405 nm (T80 UV-Vis Spectrophotometer, PG Instruments LTD, Leicester, UK) in three replicates, and the result was expressed as the mean value. The percentage of α-glucosidase inhibition was calculated according to the previously stated Equation (12).

### 3.8. Antimicrobial Activity

The nine bacterial strains were used for testing the antimicrobial activity of horehound extracts, of which *Bacillus cereus* ATCC 11778, *Staphylococcus aureus* ATCC 25923, *Bacillus subtilis* PY79, *Staphylococcus epidermidis* JR-07 and *Micrococcus luteus* JR-10 belong to Gr^+^ bacteria, while *Escherichia coli* ATCC 11775, *Pseudomonas aeruginosa* ATCC 9027, *Proteus mirabilis* ATCC 12453 and *Klebsiella pneumoniae* ATCC 31488 belong to Gr^−^ bacteria. Additionally, two yeast strains, *Saccharomyces cerevisiae* ATCC 9763 and *Candida albicans* ATCC 10231, were used for determining antifungal activity. The microdilution method was applied according to the CLSI protocol [39]. Cultures were grown overnight at 37 °C (i.e., 30 °C for *Bacillus* and *Saccharomyces*) on nutrient agar for bacteria and Sabouraud maltose agar for yeasts. The antimicrobial activity of each extract was tested in duplicate for each strain.

### 3.9. Design of Experiments

To set up experimental conditions suitable to ensure an exhaustive extraction of horehound, a Box–Behnken experimental design (BBD) approach, with three factors at three levels, was adopted. In both UAE and MAE, the design consisted of seventeen randomized runs with five replicates at the central point. The investigated UAE variables were temperature (40, 60 and 80 °C), extraction time (40, 60 and 80 min) and ultrasonic power (24, 42 and 60 W/L), while the investigated MAE parameters were ethanol concentration (30, 50 and 70%), extraction time (15, 25 and 35 min) and microwave power (400, 600 and 800 W). Unfortunately, temperature in MAE was not selected as variable due to the technical limitations of the microwave oven. Each of the coded variables was forced to range from −1 to 1 in order to normalize the parameters so that they would all affect the response more evenly, and the units of the parameters are irrelevant [40]. The natural and coded values of independent UAE and MAE variables used in BBD are presented in Table 7. 

The response variables were fitted to the commonly used second-order polynomial model (Equation (13)) [41]:

(13) $$Y=\beta_{0}+\overset{2}{\underset{i-1}{\sum }}\beta iXi+\overset{2}{\underset{i-1}{\sum }}\beta iiXi²+\overset{2}{\underset{i<j=1}{\sum }}\beta ijXiXj$$

where, *Y* represents the response variable, *X_i_* and *X_j_* are the independent variables affecting the response, and *β*_0_, *β_i_*, *β_ii_*, and *β_ij_* are the regression coefficients for mean, linear, quadratic and cross-product terms. Optimal extraction conditions were determined considering total phenols and total flavonoid contents, and the antioxidant capacity was expressed by two in vitro assays (DPPH and FRAP), while selection of optimal conditions was based on desirability function, D (Derringer and Suich, 1980). Design-Expert v.7 Trial (Stat-Ease, Minneapolis, MN, USA) was used for multiple linear regression analysis. The fitness of the polynomial model equation is expressed by the coefficient of determination (*R^2^*) and its statistical significance was confirmed by F-test at a probability (*p*) of 0.10 or 0.05. In order to verify the obtained empirical models, validation was performed by extracts preparation at optimized conditions. The confidence interval (95%) of the predicted values was compared with the experimentally observed Y, TP, TF, IC_50_ and EC_50_ in the optimized extracts.

## 4. Conclusions

The UAEopt proved to be slightly more abundant in phenolic acids compared to MAEopt, with a higher content of chlorogenic acid, as well as the flavonoid quercetin. The UAEopt shows the highest inhibitory potential, the order of magnitude of generic antidiabetic drug acarbose, for both α-amylase and α-glucosidase, which can potentially be explained by the presence of chlorogenic acid and quercetin, which were not identified in the macerates. The most sensitive bacterial strain to UAEopt is *B. cereus*. In addition, *Saccharomyces* is multiple times more sensitive to the action of UAEopt compared to *Candida albicans*, while inhibition of *S. cerevisiae* growth requires about four times higher MAC concentration. Thus, UAEopt, obtained at temperature of 73.6 °C, extraction time of 40 min and ultrasound power of 30.3 W/L, is the most superior extract in terms of antihyperglycaemic and antimicrobial activities. The future perspective of this study would be to tranform the liquid UAEopt extract into a more stable powder form and incorporate it as a component in an oral antihyperglycaemic drug. In respect to industrial applications, ultrasound-assisted extraction can be scaled-up in a more straightforward manner compared to microwave-assisted extraction.

## Figures and Tables

**Table 1:** BBD matrix with natural UAE parameters and experimentally observed values of TP, TF, IC_50_ and EC_50_ values.

Independent Variables				Investigated Responses				
Run Order	Temperature (°C)	Extraction Time (min)	Ultrasonic Power (W/L)	Y (mg/mL)	TP (mg GAE/g)	TF (mg CE/g)	IC_50_ (mg/mL)	EC_50_ (mg/mL)
1	60	60	42	15.92	85.10	50.42	0.0172	0.0593
2	60	40	60	16.02	87.66	52.40	0.0180	0.0566
3	80	40	42	18.48	99.30	53.19	0.0218	0.0575
4	60	60	42	15.76	76.36	49.84	0.0178	0.0591
5	60	80	60	17.42	80.10	48.58	0.0177	0.0576
6	40	80	42	15.16	78.94	44.97	0.0215	0.0561
7	80	60	60	20.08	92.39	50.93	0.0256	0.0636
8	40	40	42	12.91	76.00	49.03	0.0199	0.0480
9	60	60	42	16.90	82.45	44.67	0.0178	0.0557
10	40	60	60	15.35	73.66	49.08	0.0207	0.0505
11	60	60	42	15.95	82.15	51.10	0.0164	0.0562
12	60	40	24	15.19	79.85	50.51	0.0170	0.0524
13	80	60	24	17.95	94.57	44.59	0.0193	0.0566
14	60	80	24	17.15	77.58	49.15	0.0222	0.0569
15	40	60	24	13.61	83.89	50.35	0.0197	0.0427
16	80	80	42	19.11	90.00	48.25	0.0217	0.0568
17	60	60	42	16.22	86.99	52.97	0.0218	0.0606

**Table 2:** BBD matrix with natural MAE parameters and experimentally observed values of TP, TF, IC_50_ and EC_50_ values.

Independent Variables				Investigated Responses				
Run Order	Extraction Time (min)	Ethanol Conc. (%)	Microwave Power (W)	Y (mg/mL)	TP (mg GAE/g)	TF (mg CE/g)	IC_50_ (mg/mL)	EC_50_ (mg/mL)
1	35	50	400	18.29	107.08	56.05	0.0216	0.0671
2	15	50	400	18.20	106.85	57.97	0.0197	0.0635
3	15	70	600	13.74	102.58	65.54	0.0192	0.0475
4	25	30	400	18.26	93.33	59.58	0.0188	0.0630
5	15	50	800	18.70	86.05	54.70	0.0171	0.0636
6	25	50	600	17.26	92.89	57.92	0.0198	0.0659
7	35	50	800	20.36	81.94	52.10	0.0275	0.0651
8	25	50	600	19.07	109.13	52.42	0.0159	0.0726
9	25	50	600	19.65	82.28	49.98	0.0212	0.0677
10	25	70	800	16.36	94.00	57.30	0.0240	0.0590
11	25	30	800	22.30	94.36	49.76	0.0164	0.0776
12	35	30	600	22.61	86.69	53.46	0.0129	0.0790
13	25	50	600	18.95	89.36	52.96	0.0186	0.0657
14	25	70	400	14.64	96.04	62.78	0.0195	0.0528
15	15	30	600	20.69	82.29	50.93	0.0178	0.0735
16	35	70	600	14.33	117.58	65.80	0.0259	0.0445
17	25	50	600	18.16	116.94	54.58	0.0175	0.0633

**Table 3:** ANOVA of the fitted second-order polynomial models.

Ultrasound Assisted Extraction		Microwave Assisted Extraction	
**Extraction Yield**			
	***p*-value**		***p*-value**
**Model**	0.0002 *	**Model**	0.0054 *
X_1_-Temperature	<0.0001 *	X_1_-Extraction time	0.2208
X_2_-Extraction time	0.0034 *	X_2_-Ethanol concentration	0.0001 *
X_3_-Ultrasonic power	0.0105 **	X_3_-Microwave power	0.0339 **
X_1_ × X_2_	0.1566	X_1_ × X_2_	0.5696
X_1_ × X_3_	0.7143	X_1_ × X_3_	0.5051
X_2_ × X_3_	0.5972	X_2_ × X_3_	0.3367
X_1_^2^	0.2930	X_1_^2^	0.8479
X_2_^2^	0.9427	X_2_^2^	0.1481
X_3_^2^	0.2470	X_3_^2^	0.7798
**Lack of fit**	0.3136	**Lack of fit**	0.2446
**Total phenols content**			
	***p*-value**		***p*-value**
**Model**	0.0423 **	**Model**	0.0024 *
X_1_-Temperature	0.0013 *	X_1_-Extraction time	0.6941
X_2_-Extraction time	0.2320	X_2_-Ethanol concentration	0.0008 *
X_3_-Ultrasonic power	0.8710	X_3_-Microwave power	0.0006 *
X_1_ × X_2_	0.2040	X_1_ × X_2_	0.9955
X_1_ × X_3_	0.3880	X_1_ × X_3_	0.6239
X_2_ × X_3_	0.5638	X_2_ × X_3_	0.4306
X_1_^2^	0.0931 ***	X_1_^2^	0.0238 **
X_2_^2^	0.7544	X_2_^2^	0.0246 **
X_3_^2^	0.7782	X_3_^2^	0.0137 **
**Lack of fit**	0.3594	**Lack of fit**	0.0692 ***
**Total flavonoids content**			
	***p*-value**		***p*-value**
**Model**	0.0195 **	**Model**	0.0396 **
X_1_-Temperature	0.6171	X_1_-Extraction time	0.8377
X_2_-Extraction time	0.0123 **	X_2_-Ethanol concentration	0.0024 *
X_3_-Ultrasonic power	0.1637	X_3_-Microwave power	0.0281 **
X_1_ × X_2_	0.2324	X_1_ × X_2_	0.7070
X_1_ × X_3_	0.0047 *	X_1_ × X_3_	0.9099
X_2_ × X_3_	0.2107	X_2_ × X_3_	0.4765
X_1_^2^	0.0145 **	X_1_^2^	0.4200
X_2_^2^	0.6597	X_2_^2^	0.0485 **
X_3_^2^	0.1588	X_3_^2^	0.7988
**Lack of fit**	0.4961	**Lack of fit**	0.0590 ***
**IC_50_ value**			
	***p*-value**		***p*-value**
**Model**	0.0006 *	**Model**	0.0432 **
X_1_-Temperature	0.1235	X_1_-Extraction time	0.0605 ***
X_2_-Extraction time	0.2852	X_2_-Ethanol concentration	0.0091 *
X_3_-Ultrasonic power	0.0512 ***	X_3_-Microwave power	0.4196
X_1_ × X_2_	0.2903	X_1_ × X_2_	0.0367 **
X_1_ × X_3_	0.0029 *	X_1_ × X_3_	0.1003
X_2_ × X_3_	0.0054 *	X_2_ × X_3_	0.1696
X_1_^2^	<0.0001 *	X_1_^2^	0.3658
X_2_^2^	0.1935	X_2_^2^	0.5369
X_3_^2^	0.0421 **	X_3_^2^	0.1437
**Lack of fit**	0.2875	**Lack of fit**	0.3580
**EC_50_ value**			
	***p*-value**		***p*-value**
**Model**	0.0458 **	**Model**	0.0460 **
X_1_-Temperature	0.0037 *	X_1_-Extraction time	0.6595
X_2_-Extraction time	0.1828	X_2_-Ethanol concentration	0.0010 *
X_3_-Ultrasonic power	0.0559 ***	X_3_-Microwave power	0.2885
X_1_ × X_2_	0.1967	X_1_ × X_2_	0.4941
X_1_ × X_3_	0.8936	X_1_ × X_3_	0.8581
X_2_ × X_3_	0.5826	X_2_ × X_3_	0.4932
X_1_^2^	0.0792 ***	X_1_^2^	0.4893
X_2_^2^	0.7284	X_2_^2^	0.2220
X_3_^2^	0.2764	X_3_^2^	0.9648
**Lack of fit**	0.1248	**Lack of fit**	0.0708 ***

* highly significant (*p* < 0.01); ** significant (0.01 < *p* < 0.05); *** moderately significant (0.05 < *p* < 0.10); ***p*-value**, or probability value, is a number describing how likely it is that your data would have occurred by random chance; regression model exhibits **lack-of-fit** (*p* < 0.01) when it fails to adequately describe the functional relationship between the experimental factors and the response variable.

**Table 4:** HPLC-DAD analyses of ethanol, water, UAEopt and MAEopt horehound extracts.

Sample	Content (μg/mL)						
	Ferulic Acid	*p*-Coumaric Acid	Caffeic Acid	Rutin	Hyperoside	Chlorogenic Acid	Quercetin
96% EtOH	5.05	6.67	6.43	20.41	2.71	n.d.	n.d.
70% EtOH	21.38	23.89	13.86	190.43	13.05	n.d.	n.d.
50% EtOH (MAC)	35.86	30.60	18.10	236.93	25.58	n.d.	n.d.
30% EtOH	25.02	29.49	35.26	87.51	11.39	n.d.	n.d.
MAC-W	8.39	tr	tr	30.63	5.78	n.d.	n.d.
UAEopt	1.34	4.75	0.26	49.59	19.06	33.11	34.88
MAEopt	1.07	4.26	tr	34.01	14.71	23.23	30.05

Abbrevation: tr, traces; n.d., not detected.

**Table 5:** Estimated optimal UAE and MAE parameters.

Extraction Method	Optimized Conditions	Predicted Responses	Observed Responses
**UAE**		Y = 16.93%	Y = 16.90%
	Temperature: 73.6 °C	TP = 91.48 mg GAE/g	TP = 109.62 mg GAE/g
	Extraction time: 40 min	TF = 50.07 mg CE/g	TF = 53.36 mg CE/g
	Ultrasonic power: 30.3 W/L	IC_50_ = 0.0181 mg/mL	IC_50_ = 0.0189 mg/mL
		EC_50_ = 0.0564 mg/mL	EC_50_ = 0.0623 mg/mL
**MAE**		Y = 15.76%	Y = 16.66%
	Ethanol concentration: 63.8%	TP = 110.04 mg GAE/g	TP = 110.26 mg GAE/g
	Extraction time: 15 min	TF = 62.45 mg CE/g	TF = 54.86 mg CE/g
	Microwave power: 422 W	IC_50_ = 0.0191 mg/mL	IC_50_ = 0.0180 mg/mL
		EC_50_ = 0.0545 mg/mL	EC_50_ = 0.0555 mg/mL

**Table 6:** Antimicrobial activity (MIC and MBC values) of horehound extracts.

Strain	MIC Value (mg/mL)			MBC Value (mg/mL)		
	MAEopt	MAC	UAEopt	MAEopt	MAC	UAEopt
*Escherichia coli* ATCC 11775	25.00	25.00	25.00	25.00	50.00	25.00
*Klebsiella pneumoniae* ATCC 31488	25.00	25.00	12.50	25.00	25.00	25.00
*Proteus mirabilis* ATCC 12453	12.50	25.00	25.00	25.00	50.00	25.00
*Pseudomonas aeruginosa* ATCC 9027	25.00	25.00	25.00	25.00	50.00	50.00
*Staphylococcus aureus* ATCC 25923	12.50	12.50	12.50	25.00	25.00	25.00
*Staphylococcus epidermidis* JR-07	6.25	12.50	12.50	25.00	50.00	25.00
*Micrococcus luteus* JR-10	12.50	12.50	12.50	50.00	50.00	50.00
*Bacillus cereus* ATCC 11778	3.13	6.25	3.13	6.25	6.25	6.25
*Bacillus subtilis* PY79	12.50	12.50	12.50	>50.00	>50.00	>50.00
*Candida albicans* ATCC 10231	12.50	6.25	12.50	25.00	25.00	25.00
*Saccharomyces cerevisiae* ATCC 9763	≤0.05	0.20	≤0.05	≤0.05	1.56	≤0.05

**Table 7:** Experimental domain with natural and coded values of independent variables.

Extraction Method	Independent Variable	Factor Levels		
		−1	0	1
	Temperature (°C)	40	60	80
**UAE**	Extraction time (min)	40	60	80
	Ultrasonic power (W/L)	24	42	60
	Extraction time (min)	15	25	35
**MAE**	Ethanol concentration (%)	30	50	70
	Microwave power (W)	400	600	800

## Data Availability

Not applicable.
